# Items

**Published:** 1900-06

**Authors:** 


### ITEM.

Mr. W. B. Saunders, the well-known medical book publisher of Philadelphia, announces the final accomplishment of a step that he has long had in mind. Feeling that the growth of the business to its present large proportions has been due, not alone to his own exertions, but quite as much to the efficient coöperation of a number of his employees, he has decided to give recognition to such service by associating with himself in business, under the firm name of W. B. Saunders & Company, Mr. F. L. Hopkins, Manager of the Subscription Department and Mr. T. F. Dagney, Manager of the Publication Department. These gentlemen have been connected with the establishment almost from its inception, and to their capable management of their respective departments Mr. Saunders attributes much of the success that has attended his efforts.

Mr. Saunders believes that this action will strengthen the position of the house in the eyes of the medical profession, as it will secure a permanence of organisation that will ensure the perpetuation of the business. Besides this, it will obviate the disadvantages incident to a large business that rests entirely upon the shoulders of one person, by permanently attaching to the house those whose ability and experience have contributed in bringing the business to its present state of prosperity.

The subscription and publication departments will be conducted as heretofore. The trade book department will be under the management of Mr. W. D. Watson, whose connection with the house has extended over the past eight years, and who has demonstrated his ability to manage that department with efficiency and success.

